# Dynamic changes in gene alterations during chemotherapy in metastatic castrate resistant prostate cancer

**DOI:** 10.1038/s41598-022-08520-6

**Published:** 2022-03-18

**Authors:** Winston Tan, Tiantian Zheng, Amy Wang, Joanna Roacho, Seng Thao, Pan Du, Shidong Jia, Jianjun Yu, Bonnie L. King, Manish Kohli

**Affiliations:** 1 Department of Medicine, Mayo Clinic, Jacksonville, USA; 2 Predicine, Inc., 3555 Arden Road, Hayward, CA 94545 USA; 3 Division of Oncology, Department of Medicine, Jack R. and Hazel M. Robertson Presidential Endowed Chair, Huntsman Cancer Institute, University of Utah, 2000 Circle of Hope Dr. Rm. 4263, Salt Lake City, UT 84112 USA

**Keywords:** Cancer genomics, Metastasis, Cancer, Prostate cancer, Molecular medicine

## Abstract

Docetaxel chemotherapy is a standard treatment option for metastatic castrate resistant prostate cancer (mCRPC) patients. To date, the genomic perturbations underlying the emergence of resistance in mCRPC patients during chemotherapy treatment have not been fully characterized. Previous studies have established that *AR*, *TP53*,* RB1* and *PTEN* gene alterations are frequent at this stage of progression and that *TP53*,* RB1* and *PTEN*, but not *AR* alterations are associated with poor outcome. However, the clonal dynamics of these key driver cancer genes during chemotherapy in mCRPC patients have not been described. Toward this goal, we performed a retrospective analysis of serially profiled cell-free DNA (cfDNA) alterations in blood samples collected from mCRPC patients before and after starting chemotherapy who were followed for response and clinical outcomes. While *AR* alterations and measures of mutational load were significantly reduced in patients with stable or decreased PSA levels after 3 cycles of chemotherapy, reductions in *RB1*,* TP53* and *PTEN* alterations were relatively modest, which may represent the persistence of a clonal signature associated with the emergence of treatment-induced lineage plasticity (TILP) underlying resistance. The ability to monitor these driver gene clonal dynamics during chemotherapy may have utility in the clinical setting.

## Introduction

Prostate cancer (PCa) accounted for greater than 34,000 deaths in US males^1^ and over 325,000 deaths world-wide^2^ in 2021, with nearly all cancer-related mortality occurring in the metastatic state. The initial clinical management of metastatic prostate cancer is based on disruption of the androgen receptor (AR) signaling axis, and prior to 2015 this was achieved through the use of single agent androgen deprivation therapy (ADT). However, the clinical landscape has evolved rapidly and now includes treatment regimens based on a combinatorial approach of ADT with multiple drugs^3^. While ADT based combination treatments slow progression of disease, they inevitably fail in most patients with the emergence of castrate resistance^4^. Current standard-of-care treatment options for this stage of prostate cancer progression include different chemotherapeutic agents such as docetaxel and cabazitaxel, the androgen receptor signal inhibitors (ARSIs) abiraterone and enzalutamide, and radionuclides (Radium-223)^3–7^. However, there are currently no approved guidelines for choosing between these multiple approved drug treatment options^8^. Biomarkers are urgently needed to define which patients will benefit from the different treatment options in a first line setting, define the optimal sequence and combination of therapies, and track the response and emergence of treatment resistance. Toward that end, a variety of biomarkers including PSA^8^, alkaline phosphatase (ALP)^8^, βIII-tubulin^9^, CTC numbers^10,11^ and molecular alterations including AR-V7 splice variants^12–17^, plasma ctDNA alterations^18–26^ and plasma cell-free DNA (cfDNA)^27–29^ have been evaluated to predict and monitor response. However, none of these biomarkers have been clinically validated for choosing between therapies and their remains a critical need to understand the molecular profile and driver gene clonal dynamics during chemotherapy in order to maximize treatment accuracy and benefit.

In the present study, we used a targeted NGS-based liquid biopsy approach to profile serially obtained plasma samples collected prospectively and analyzed retrospectively in a cohort of mCRPC patients before and after undergoing docetaxel chemotherapy. Here we describe the dynamics of key driver gene alterations during chemotherapy in association with treatment response and outcome, which may underlie the emergence of resistance and impact survival.

## Materials and methods

### Patient enrollment and sample collection

The current study was approved by the IRB of the Mayo Clinic. Metastatic prostate cancer patients were prospectively enrolled at a single tertiary-level cancer center (Mayo Clinic, Rochester, MN) after obtaining informed consent in an Institutional Review Board-approved study (MC IRB # 09-001889: “Study of Molecular Circulatory Biomarkers in Hormone Sensitive and Castration Recurrent Prostate Cancer”). All methods were carried out in accordance with relevant guidelines and regulations, and all experimental protocols were approved by the above-cited IRB-approved study at the Mayo Clinic. Serial blood samples were collected between September 2009 and January 2013 as previously described^30–32^. Enrolled patients were followed for outcomes until June 2021. Methods for extracting cell-free (cfDNA) and germline DNA (gDNA) have been previously published^33^ and are also detailed in Supplementary Methods.

### Next generation sequencing (NGS) methods

The details of library preparation, amplification, capture and sequencing, as well as the analysis of sequencing data have been previously described^33^ and are also provided in Supplementary Methods. Briefly, libraries were constructed using extracted cfDNA and fragmented gDNA, amplified by PCR and then subjected to hybrid capture using the PredicineCARE panel (Supplementary Table 1). Sequencing data were analyzed using Predicine’s in-house analysis pipeline encompassing the initial analysis of raw sequencing data base call files through variant calling. Details on variant calling, copy number variation (CNV) estimation methods, and the calculation of circulating tumor DNA (ctDNA) fraction and plasma Tumor Mutational Burden (pTMB) have been previously reported^33^. Total alteration counts represent the total number of single nucleotide variants (SNVs) and CNVs detected per sample across all genes in the PredicineCARE panel. Top alteration counts represent the total number of SNVs and CNVs detected per sample across a set of top 32 most frequently altered genes. Variant allele frequencies (VAFs), representing the fraction of altered/total alleles, were normalized to ctDNA fractions.

### Statistical analysis methods

Survival analysis was performed to evaluate associations of ctDNA-based alterations measured prior to the commencement of chemotherapy in the mCRPC state with overall survival (OS). This extends our pre-defined and published analysis reported after a median follow up for 94.67 months at a data-freeze date of June 2018^33^, to June 2021 for a median follow-up of 130.6 months^33^. OS was calculated from the date of first sample collection to the date of death or to the last follow-up for alive patients at the time of the cutoff date of the analysis. We also performed a retrospective exploratory analysis to determine clonal dynamic changes with treatment, in serially captured and previously sequenced cfDNA samples from mCRPC patients^33^. Kaplan–Meier plots were used to show survival and the log-rank test was used for comparing survival differences between groups. Associations of variables with OS were also evaluated by univariate and multivariate analyses using Cox proportional hazards regression and the log-rank test. Scaled Schoenfeld residuals and deviance residuals with time were examined to ensure the validity of the Cox regression assumptions. To account for multiple hypothesis testing, adjusted P-values using the Benjamini and Hochberg procedure are reported^34^. Age, ctDNA fraction, Gleason score and alkaline phosphatase levels were included in the multivariate analysis of OS in the mCRPC state. To dichotomize the patient cohorts for certain variables, the upper quartile cutoff was used for ctDNA fraction, and the median cutoff was used for alkaline phosphatase levels. A Gleason Score of 8 or above was defined as high, and a score of less than or equal to 7 was defined as low. Paired Wilcoxon tests were performed to examine differences of ctDNA, pTMB, total number of alterations, total SNV counts, total CNV counts and VAFs between paired patient samples collected before and after three months of chemotherapy. McNemar’s test was used to evaluate differences in the frequency of alterations in specific genes across paired patients before and during chemotherapy. The Wilcoxon test was used to examine differences of ctDNA fraction, pTMB and total alteration counts in unpaired patients before vs. during chemotherapy. The Fisher’s exact test was used to evaluate differences in the frequency of mutations across specific genes in unpaired patients before and during chemotherapy. All statistical analyses were performed using R version 3.5.3 and all tests of statistical significance were two-tailed with a significance set at *p* ≤ 0.05.

## Results

### Patient enrollment, study design and treatment groups

**Figure 1:**
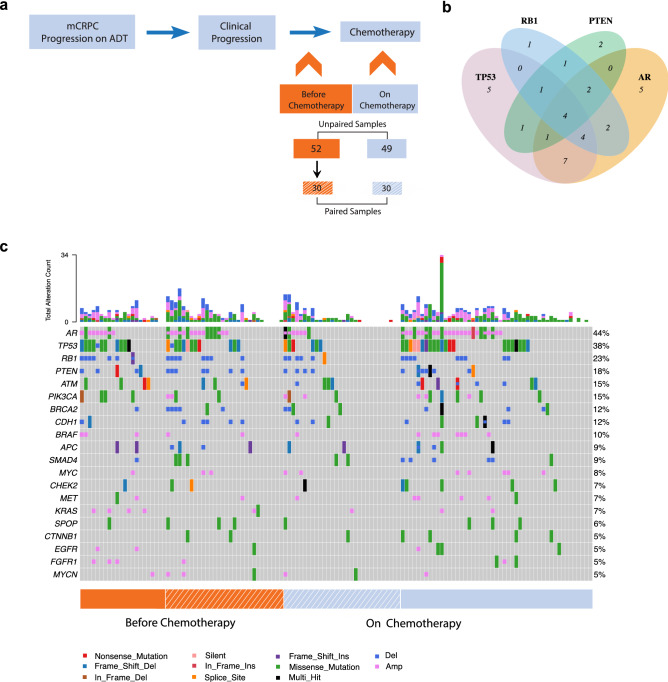
Landscape of somatic alterations detected in mCRPC patients before and during chemotherapy. (**a**) Plasma samples were collected from mCRPC patients following clinical progression on androgen deprivation therapy (ADT), prior to the initiation of chemotherapy (n = 52). Thirty of these patients were serially sampled during chemotherapy to create a subset of paired samples (before chemotherapy, n = 30; after 3–4 chemotherapy cycles, n = 30). Forty-nine samples were collected from an independent group of patients following 3–4 chemotherapy cycles (n = 49). (**b**) Venn diagram illustrating the overlap of the 4 most frequently altered genes (*AR*,* TP53*,* RB1* and *PTEN*) detected at baseline in individual patients. (**c**) Heatmap of somatic alterations including SNVs and CNVs detected across all samples collected before and after 3–4 cycles of chemotherapy. The percentage of alterations detected in each gene across all patient samples is shown to the right of the heatmap. The total number of alterations detected in each patient sample is shown in the bar graph above the heat map. This figure was created by manuscript authors using the Adobe Illustrator 2020 (https://www.adobe.com/products/illustrator.html) (**a**), VennDiagram (version 1.7.1) (https://cran.r-project.org/web/packages/VennDiagram/index.html) (**b**), and Bioconductor (https://www.bioconductor.org/packages/release/bioc/html/maftools.html) (version 3.14) (**c**) software packages.

Three hundred and three metastatic prostate cancer patients were prospectively enrolled at the Mayo Clinic from September 2009 to January 2013, and followed until death, with a cutoff date of June 13, 2021, for analyses. This study sub-cohort is composed of 101 of the 303 patients who experienced clinical progression (defined by biochemical failure and/or the appearance of new radiographic metastases) during androgen deprivation therapy (ADT), and subsequently underwent chemotherapy for the mCRPC state. All patients with sufficient plasma sample volumes for cfDNA extraction were included. The workflow for plasma samples collected and processed for analyses from these patients is shown in Supplementary Fig. S1. Samples were collected prior to the commencement of chemotherapy, and after 3–4 chemotherapy treatments to allow adequate drug exposure (Fig. 1a). Of the 52 samples collected prior to chemotherapy, 30 patients were sampled serially a second time during chemotherapy, creating a subset of paired samples (before chemotherapy, n = 30; after at least 3 cycles of chemotherapy, n = 30). An additional 49 samples were collected from a separate cohort of mCRPC patients when they had received at least three to four cycles of chemotherapy, but did not have matched pre-chemotherapy samples (unpaired sample set, n = 49)). The demographic data for all patients (n = 101) are described in Table 1.

**Table 1:** Clinical characteristics of metastatic castrate resistant prostate cancer (mCRPC) patients undergoing chemotherapy following clinical progression on androgen deprivation therapy (ADT).

	Before Chemotherapy (n = 52)		During Chemotherapy
	Unpaired group, n = 22	Paired group, n = 30	Unpaired group, n = 49
**Total patients, no.**	24	31	49
**Patients with analyzable NGS data (N)**	22	30	49
Age in years at the time specimen collection, median (range)	70 (50–86)	72.5 (54–87)	73 (44–87)
**Gleason Score at ID, no.**			
≤ 7	9	11	25
≥ 8	12	16	20
Unknown	1	3	4
**Clinical TNM staging at ID, no.**			
T1	1	1	1
T2	8	8	24
T3	8	14	16
T4	0	1	0
T-unknown	5	6	8
N0	6	14	15
N1	7	4	17
N-unknown	9	12	17
M0	15	23	31
M1	5	7	13
M-unknown	2	0	5
Median time to second sample collection, day (range)	–	99.5 (64, 204)	–
**PSA at time of sample collection, median ng/ml (IQR)**			
First sample	35.8 (0.24, 214)	15.9 (1.60, 612)	49.5 (0, 1500)
Second sample	N/A	24.6 (0, 800)	N/A
ALP at the time of sample collection, median (range)	118 (39.0, 605)	100 (44.0, 2190)	98.5 (48.0, 812)
**LDH at the time of sample collection, median (range)**			
First collection	201 (171, 384)	225 (137, 278)	211 (128, 1580)
Second collection	N/A	284 (153, 1130)	N/A
Patients with missing values, no.	15	18	19
**Hemoglobin at sample collection, median (range)**	12.7 [11.1, 14.2]	13.1 [8.40, 14.8]	10.6 [9.00, 13.1]
Patients with missing values, no.	10	7	42
Radical prostatectomy on ID, no.	7	19	18
Radiation alone on ID, no.	4	2	12
Radical prostatectomy and radiation on ID, no	0	0	0
Salvage local treatments after primary prostate treatments	8	11	9
Median time from initial treatments for localized stage disease to disease progression, mo (range)	15.3 (5.9, 91.9)	29.4 (3.8, 203.2)	38.6 (0.07, 145.1)
Received docetaxel after clinical progression, no	22	30	47
Median time from ADT initiation for mHSPC stage to biochemically progress to CRPC stage, mo (range)	11.9 (3.2–55.6)	18.7 (3.7–203.0)	20.3 (0.07–106.4)
Median follow-up time from date of mCRPC specimen collection to last follow up, mo (range)	127.2 (104.0, 141.5)	133.7 (107.0, 142.5)	132.0 (105.8, 140.3)
Median time to death/last follow-up for mCRPC patients, mo (range)	23.7 (3.0, 59.2)	28.1 (5.4, 125.1)	14.2 (0.2, 92.8)
Patients dead upon follow-up, no.	22	30	47

### Genomic landscape of ctDNA-based alterations across all mCRPC (paired and unpaired) patient sub-cohorts

To examine the spectrum of genomic alterations in mCRPC patients before and during chemotherapy, we extracted cell-free DNAs from patient plasma. SNVs and CNVs were profiled with a targeted NGS panel (PredicineCARE). We determined measures of ctDNA fraction, pTMB and total alteration counts across all patient samples collected before and during chemotherapy. Figure 1b shows overlap of the top 4 most frequently altered genes (*AR*,* TP53*,* RB1* and *PTEN*) in some patients. Figure 1c shows the top 20 most frequently altered genes in ≥ 5% of all study patient samples before and during chemotherapy. The most frequently altered genes across all patient samples were *AR* (44%), *TP53* (38%), *RB1* (23%), *PTEN* (18%), *ATM* (15%), *PIK3CA* (15%), *BRCA2* (12%), *CDH1* (12%) and *BRAF* (10%). These most frequently altered genes were selected for analysis of association with the overall survival for the sub-cohort of 52 patients whose initial samples were collected prior to chemotherapy.

### Patient outcomes based on somatic alterations detected before chemotherapy

To investigate the potential prognostic and predictive value of ctDNA-based alterations, we evaluated overall survival (OS) in association with cfDNA yield, ctDNA fraction, pTMB, SNVs and CNVs assessed in the sub-cohort of 52 patient samples collected prior to initiating chemotherapy. High (upper quartile) ctDNA fraction was associated with shorter OS (*p* = 0.0008) (Supplementary Fig. S2), whereas no significant reduction was observed in association with cfDNA yield or pTMB (Supplementary Fig. S3). At the univariate level, the presence of a CNV or SNV in *RB1* (*p* < 0.0001), *TP53* (*p* = 0.003) *AR* (*p* = 0.003), *PTEN* (*p* < 0.0001) or *CDH1* (*p* = 0.0005) (Supplementary Fig. S2), was significantly associated with shorter OS. In multivariate analyses (Supplementary Table 2), after adjustment for age, ctDNA fraction, Gleason Score and alkaline phosphatase levels, the presence of a SNV *or* CNV in the *TP53* (*p* = 0.009), *CDH1* (*p* = 0.007), *RB1* (*p* = 0.01), or *PTEN* (*p* = 0.01) genes independently remained significantly associated with shorter OS. Genes significantly associated with OS before and after adjustment for multiple clinical and genomic covariables are listed in Supplementary Table 2.

*Comparison of ctDNA-based profiles across the unpaired patient sub-cohorts.* To compare the landscape of ctDNA alterations in mCRPC chemotherapy-naïve patients vs. patients after the initiation of chemotherapy treatment, we analyzed genomic profiles across the unpaired patient sub-cohorts. No differences were detected in ctDNA-based profiles between the independent groups of patients who had not started chemotherapy vs. patients who had received 3–4 cycles of chemotherapy (Supplementary Fig. 4).

*Pharmacodynamic changes in ctDNA-based alterations during chemotherap*y *in the paired patient sub-cohort*. In the paired cohort, to identify changes associated with PSA-based response to chemotherapy, we compared ctDNA profiles across the set of patients with serial samples collected before and after 3–4 cycles of treatment. For associating changes with clinical outcomes in this sub-cohort of paired samples from the same patient, we classified patients into two groups based on PSA response after 3–4 treatments. Patients exhibiting a 25% or greater increase in PSA level at the time of the serial sample collection after three to four chemotherapy treatments were classified as 3-month PSA non-responders, and the remaining patients were classified as the “PSA stable to decrease” group who continued on the same chemotherapy in line with standard of care decided by the treating physician^35^.

**Figure 2:**
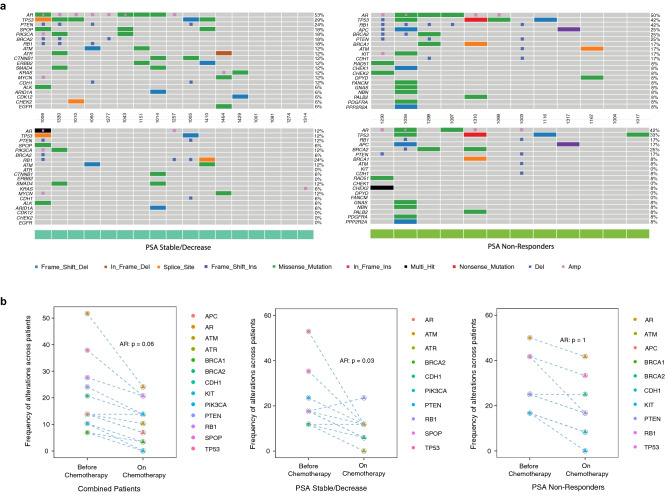
Pharmacodynamic changes in genomic alterations in response to chemotherapy. (**a**) Heatmaps of somatic alterations detected in paired mCRPC samples collected before (top) and after (bottom) 3–4 cycles of chemotherapy from patients who were classified for response to chemotherapy on the basis of PSA levels at the time of second sample collection. Patients exhibiting stable or decreased PSA levels were classified as “PSA Stable/Decrease” and patients exhibiting increased PSA levels as “PSA Non-Responders”. The frequency of alterations observed in a given gene across all patients is listed to the right of the heatmaps. (**b**) Graphical representation of heatmap data for the top 10 most frequently altered genes across all paired patients combined (n = 29), patients with stable or decreasing PSA (n = 17) and patients with increasing PSA levels (n = 12) before and after 3–4 cycles of chemotherapy. The frequency of alterations detected in the *AR* gene was significantly reduced after chemotherapy in the group of patients with stable/decreasing PSA (*p* = 0.03). Comparisons were made using the McNemar’s test with significance set at *p* ≤ 0.05. The heatmaps in this figure were created by manuscript authors using the Bioconductor ComplexHeatmap software package (version 3.14) (https://www.bioconductor.org/packages/release/bioc/html/ComplexHeatmap.html).

**Figure 3:**
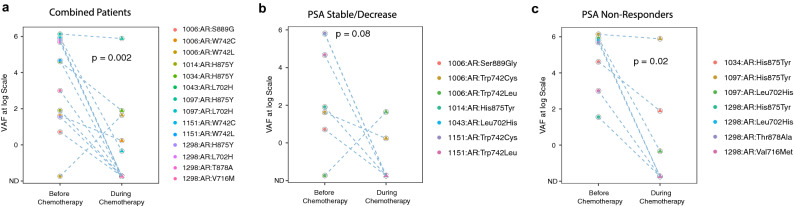
Pharmacodynamic changes in *AR* variant allelic frequencies (VAFs) in response to chemotherapy. Comparisons were made in patients who were classified for response to chemotherapy on the basis of PSA levels at the time of second sample collection following 3–4 cycles of treatment. Patients exhibiting stable or decreased PSA levels were classified as “PSA Stable/Decrease” and patients exhibiting increased PSA levels as “PSA Non-Responders”. (**a**) A significant reduction in the median VAF level following chemotherapy was observed in the group including all patients (n = 29, *p* = 0.002). (**b,c)** Reductions in median VAF levels were also observed in patients with stable or decreased PSA levels (n = 17, *p* = 0.08) and patients with increased PSA levels (n = 12, *p* = 0.02), although due to the small number of *AR* SNVs in each group, distinct differences could not be detected between the two groups. Comparisons were made using the paired Wilcoxon test with significance set at *p* ≤ 0.05.

**Figure 4:**
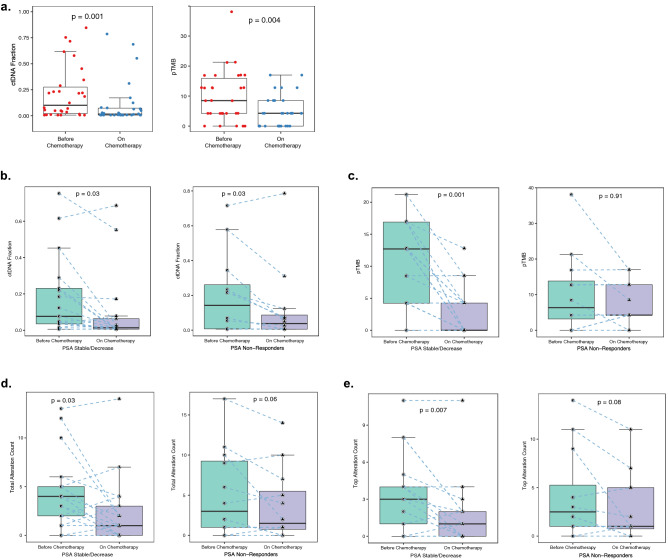
Pharmacodynamic changes in ctDNA fraction, pTMB and total alteration counts in response to chemotherapy. Comparisons were made in patients who were classified for response to chemotherapy on the basis of PSA levels at the time of second sample collection following 3–4 cycles of treatment. Patients exhibiting stable or decreased PSA levels were classified as “PSA Stable/Decrease” and those exhibiting increased PSA levels as “PSA Non-Responders”. (**a**) Median ctDNA fraction (*p* = 0.001) and pTMB (*p* = 0.004) were significantly reduced in the group of combined patients (n = 29) after 3–4 cycles of docetaxel. (**b**) Similar significant reductions in ctDNA fraction were observed for the “PSA Stable/Decrease” patients (n = 17, *p* = 0.03) and the “PSA Non-Responder” patients (n = 12, *p* = 0.03). (**c**) Median pTMB was significantly reduced in the “PSA Stable/Decrease” patients (*p* = 0.001) but not the “PSA Non-Responder” patients (*p* = 0.91). (**d**) Similar reductions were observed in median total alteration counts for the “PSA Stable/Decrease” patients (*p* = 0.03) and the “PSA Non-Responder” patients (*p* = 0.06). When this comparison was restricted to the top 32 most altered genes, values were significantly reduced in the “PSA Stable/Decrease” patients (*p* = 0.007), and modestly in the “PSA Non-Responder” patients (*p* = 0.08). Comparisons were made using the paired Wilcoxon test with significance set at *p* ≤ 0.05.

The landscape of ctDNA-based alterations across the paired patient sample sets collected from both groups is presented in heatmaps in Fig. 2a. Figure 2b shows that the frequency of alterations in most of the top-mutated genes trended lower after 3–4 cycles of chemotherapy, but most of these trends were not significant. The one exception to this trend was a non-significant increase in the frequency of *RB1* alterations in the patients with stable or decreased PSA levels on chemotherapy. Notably, the only gene alteration to decrease significantly after chemotherapy in the paired samples was *AR.* After 3–4 cycles of treatment the frequency of *AR* alterations decreased substantially in patients with stable or decreased PSA at 3-months (53–12%; *p* = 0.03) but not in patients in the 3-month PSA non-responder group (50–42%; *p* = 1). The high frequency of AR alterations across patients enabled additional comparison of individual *AR* VAFs, which were also significantly reduced during chemotherapy across all patients (*p* = 0.002) (Fig. 3a). Reductions in VAF levels were also observed in patients with stable or decreased PSA levels (*p* = 0.08) and patients with increased PSA levels (*p* = 0.02), although due to the small number of *AR* SNVs in each group, distinct differences could not be detected between the two groups (Fig. 3b,c). Both ctDNA fraction (*p* = 0.001) and pTMB (*p* = 0.004) were also significantly reduced across all serial samples (Fig. 4a). While ctDNA fraction reductions were similar in both 3-month PSA groups (Fig. 4b), the reduction in pTMB was significantly greater in patients with stable or decreased PSA levels (*p* = 0.001) than in non-responders (*p* = 0.91) (Fig. 4c). Total alteration counts (including SNVs and CNVs) were also reduced in serial samples after chemotherapy in patients with stable or decreased PSA levels (*p* = 0.03) and in 3-month PSA non-responders (*p* = 0.06), (Fig. 4d). When total alterations were assessed for the top 32-mutated genes only, top alteration counts were significantly reduced in patients with stable or decreased PSA levels (*p* = 0.007), but to a lesser degree in the 3-month PSA non-responders group (*p* = 0.08) (Fig. 4e). Similar comparisons focusing exclusively on SNV or CNV counts revealed significant decreases in SNV counts after chemotherapy in the patients with stable or decreased PSA levels (*p* = 0.01) but not in the PSA non-responders (*p* = 0.57). No significant reductions were observed for CNV counts in either group (Supplementary Fig. 5).

## Discussion

In this study we confirmed the association of RB1, TP53 and PTEN, but not AR gene alterations detected in mCRPC patients prior to initiating chemotherapy with poor OS. We also observed that dynamic changes in these and other gene alterations can be tracked during chemotherapy treatment, which has not been previously reported. Our results illustrate the potential utility of NGS-based sequencing to monitor the clonal evolutionary changes during treatments, which could potentially be useful for developing adaptive strategies that target the emergence of resistance. Recent therapeutic advances^36^ in the treatment of mCRPC have prolonged survival and increased quality of life, but therapeutic benefits are temporary, and further research is needed to develop genome-based biomarkers that may be used to optimize benefit and maximize treatment accuracy. Given the rapid emergence of acquired therapeutic resistance during metastatic progression, coupled with the need for molecular profiling during each new therapeutic regimen, liquid biopsy has been increasingly adopted for profiling tumor-associated alterations during clinical management. Previously, we characterized the genomic landscape of metastatic hormone sensitive prostate cancer using NGS-based liquid biopsy before and after ADT and observed changes in detectable genomic alterations under the influence of ADT^33^. These profiles revealed the gradual accumulation of genomic aberrations during progression and treatment, with the highest frequency of alterations most consistently observed in *AR*,* TP53*,* RB1*,* PTEN*,* APC* and DNA repair genes, in keeping with findings reported by others^5,24–26^. Although genomic features of this cohort have been reported previously^33,37,38^, the analyses undertaken in this study focuses on the effect of serial sequencing using a targeted and sensitive NGS panel, which has not been explored.

In this study we have characterized genomic profiles of mCRPC patients before and after initiating chemotherapy. Following treatment, we observed significant reductions in several genomic indices, including ctDNA fraction (presumably reflecting reductions in tumor cell numbers), total variant counts and pTMB. We were also able to track VAFs for individual AR mutations, which were also significantly reduced following chemotherapy. To further evaluate these indices and alterations as potential biomarkers for monitoring chemotherapy response, we also compared post-chemotherapy profiles in patients classified as on the basis of post-treatment PSA levels. This comparison revealed significantly greater reductions in *AR* alterations, pTMB and total alteration counts (for the top 32 mutated genes) in patients with stable or decreased PSA after three months of chemotherapy vs. those patients experiencing a PSA increase, suggesting that *AR* alterations and comprehensive measures of mutational load are promising candidate biomarkers of response to chemotherapeutic drugs.

We also demonstrated that the presence of *TP53*,* RB1* and *PTEN* but not *AR* alterations at baseline in mCRPC patients undergoing chemotherapy significantly defined survival outcomes. Interestingly, we observed that patients responding to systemic chemotherapy at 3-months based on PSA response alone had a notable decrease in *AR*, but not *RB1*,* TP53* or *PTEN* alterations. The frequency of *AR* alterations was significantly reduced in patients with stable or declining PSA values, with *AR* alterations found in 53% of patients before vs. 12% after chemotherapy. Based on these observations, while it is unclear if chemotherapy selectively targeted cancer cells bearing *AR* alterations, it significantly reduced them, while less robust reductions were observed for other key clonal alterations such as TP53, RB1 and PTEN. As these gene alterations may be drivers of resistance in the mCRPC state, pharmacodynamic profiling, if performed in real time in future patients, might present a clearer picture for identifying adaptive selection of therapeutic drugs. Of note, the presence of *TP53*,* RB1* and *PTEN* alterations have previously been associated with poor patient outcomes related to TILP and the generation of an aggressive neuroendocrine or adeno-neuroendocrine tumor phenotype and^39–41^. Although in our cohort we did not perform metastatic tissue biopsies to identify amphicrine prostate cancer histology, reduction in *AR* alterations in blood on serial monitoring in parallel with less dramatically altered levels of *TP53*,* RB1* and *PTEN* alterations suggest clonal dynamics consistent with the association of poor patient outcomes with these gene alterations in our study.

A primary limitation of our study includes the small size of our exploratory, retrospective analysis of patients with serial samples, which limited the comparison of clonal dynamics between patients with stable versus increasing PSA after 3 months of chemotherapy. In addition, we were not able to correlate plasma profiles with metastatic tissue sampling and limited our sequencing to a targeted panel of genes. Nevertheless, the serial NGS-based sequencing was able to detect candidate molecular biomarkers during a PSA-based response to chemotherapy and identify dynamic clonal patterns that may have relevance to monitoring for the development of TILP. These will need to be systematically determined in larger prospective cohorts, and if successful may enable the future development of adaptive therapeutic strategies.

## Supplementary Information

Supplementary Information.

Supplementary Data.

## Data Availability

The annotated sequencing data that support the findings of this study are available in an Excel file labeled “Raw Sequencing Data” in the supplementary materials.
