# Supplementary material for: Dynamic changes in gene alterations during chemotherapy in metastatic castrate resistant prostate cancer
Source: Sci Rep. 2022 Mar 18;12:4672. doi: 10.1038/s41598-022-08520-6 (PMC8933498; doi:10.1038/s41598-022-08520-6)
Supplement: Supplementary file 1 — Supplementary Information. [file 41598_2022_8520_MOESM1_ESM.pdf]

SUPPLEMENTARY INFORMATION

Dynamic changes in gene alterations during chemotherapy in metastatic castrate resistant prostate cancer

Winston Tan[1*], Tiantian Zheng[2]*, Amy Wang[2], Joanna Roacho[2], Seng Thao[2], Pan Du[2], Shidong Jia[2], Jianjun Yu[2], Bonnie L. King[2#], Manish Kohli[3#].

Author affiliations

1: Department of Medicine, Mayo Clinic, Jacksonville

2: Predicine Inc., Hayward, California, USA

3: Division of Oncology, Department of medicine, Huntsman Cancer Institute, University of Utah

*Co-first authors

#Co-Corresponding authors

*Correspondence to:

Manish Kohli MD

Professor, Division of Oncology, School of Medicine,

Jack R. and Hazel M. Robertson Presidential Endowed Chair

Huntsman Cancer Institute, Salt Lake City, UT

2000 Circle of Hope Dr. Rm. 4263

SLC UT 84112

Manish.kohli@hci.utah.edu

Bonnie L. King PhD

Associate Director of Translational Medicine

Predicine, Inc.

3555 Arden Road

Hayward, CA 94545

bking@predicine.com

TABLE OF CONTENTS Page

Supplementary Methods 3

Table S1: PredicineCARE panel 8

References 9

Supplementary Tables and Figures 10

Figure S1. Study design and workflow. 10

Figure S2. Pre-chemotherapy based somatic alterations and survival. 11

Figure S3. Pre-chemotherapy cfDNA yield, pTMB and survival. 12

Figure S4. Comparison of ctDNA profiles in the unpaired mCRPC patients. 13

Figure S5. Pharmacodynamic changes in SNVs and CNVs in response to chemotherapy. 14

Table S2: Univariate and multivariate analyses of overall survival (OS). 15

SUPPLEMENTARY METHODS

Specimen Processing Methods

Mayo Clinic collections were approved by the Mayo Clinic Institutional Review Board [IRB; 09-001889] for two serial collections. Details of patient enrollment and sample processing methods used have been published previously (1-5).

Ten milliliters of peripheral venous blood were collected into plasma separator tubes containing K2-ethylenediamine tetraacetic acid (EDTA) and 3.2% buffered sodium citrate anticoagulants. Collected blood was initially centrifuged at 3000 rpm for 10 minutes at 4 °C. The supernatant was then removed, and a second centrifugation was performed at 3000 rpm for 10 minutes. The platelet-poor plasma was fractioned into multiple aliquots for storage at -80 °C, with no aliquots undergoing freeze-thaw cycles prior to cfDNA isolation.

cfDNA and germline DNA extraction

Circulating cfDNA was extracted using a QIAamp Circulating Nucleic Acid Kit (QIAamp, Venlo, NL) from EDTA and citrate anticoagulant plasma samples. The average volume of plasma used for extraction was 2.6 mL (Range, 0.7-3.9 mL). The quantity and quality of the purified cfDNA were checked using a Qubit 3.0 Fluorometer (Thermo Fisher Scientific, Waltham, MA, USA) and Bioanalyzer 2100 (Agilent Technologies, Santa Clara, CA, USA). For samples with severe genomic contamination from peripheral blood cells, a size selection was performed with AMPure XP beads (Beckman Coulter, Brea, CA, USA) to remove large genomic fragments. Samples with a total yield

<5 ng were considered inadequate for next generation sequencing (NGS) and were removed from any further sequencing methods.

Germline DNA (gDNA) was extracted from matched peripheral blood mononuclear cells collected at the same time as plasma for a subset of patients. Up to 250 ng of gDNA were enzymatically fragmented to generate a main peak at ~250 bp and was further purified using AMPure XP beads as per the manufacturer's instructions.

Library preparation, capture, and sequencing

Five to 30ng cfDNA was used for library preparation with KAPA Biosystems Library Preparation Reagents. Briefly, the cfDNA was end-repaired before a dA-tailing process, and then ligated with proprietary Unique Molecular Identifier (UMI) adapters. Following PCR amplification, library yield was measured using the Qubit and subjected to quality control using the Bioanalyzer 2100. Samples with library yield >700ng were subjected to hybrid capture using Biotin-labeled DNA probes (Thermo Fisher Scientific, Weltham, MA, USA). In brief, the library was hybridized using the PredicineCARE NGS panel overnight and captured on Dynabeads M-270 Streptavidin (Thermo Fisher Scientific (Fisher Scientific, Weltham, MA, USA). The unbound fragments were washed away, and the enriched fragments were amplified via PCR amplifications. The purified product was checked using Bioanalyzer 2100 and loaded into the HiSeq X Ten (Illumina, San Diego, CA, USA) for NGS with paired-end 2×150 bp sequencing kits.

Analyses of NGS data generated from cfDNA

The analysis of the NGS data has been previously published in great detail (5). We briefly summarize these methods here.

NGS data were analyzed using the Predicine DeepSea NGS analysis pipeline, which starts from the raw sequencing data (BCL files) and outputs the final mutation calls. Briefly, the pipeline first performed adapter trim, barcode checking, and correction. Cleaned paired FASTQ files were aligned to the human reference genome build hg19 using the BWA alignment tool. Consensus bam files were then derived by merging paired-end reads originated from the same molecules (based on mapping location and unique molecular identifiers) as single strand fragments. Single strand fragments from the same double strand DNA molecules were further merged as double stranded. By using the error suppression method described in (6), both sequencing and PCR errors were mostly corrected during this process.

Candidate variants were called by comparing with local variant background (defined based on plasma samples from healthy donors and historical data). Variants were further filtered by log-odds (LOD) threshold (7), base and mapping quality thresholds, repeat regions and other quality metrics. Specific criteria for calling candidate somatic mutations for this cohort have been described previously (5). Candidate somatic mutations were further filtered on the basis of gene annotation to identify those occurring in protein-coding regions. Intronic and silent changes were excluded, and mutations resulting in missense mutations, nonsense mutations, frameshifts, or splice site alterations were retained. Mutations annotated as benign or likely benign in the ClinVar database were also filtered. Finally, hematopoietic expansion-related variants that have

been previously described (8-10), including DNMT3A, ASXL1, TET2, and specific alterations within ATM (residue 3008), GNAS (residue 201, 202), or JAK2 (residue 617) were also excluded.

DNA rearrangements were detected by identifying the alignment breaking points on the basis of the consensus sequence from the BAM files, before the consensus step. Suspicious alignments were filtered according to repeat regions, local entropy calculation, and similarity between reference and alternative alignments.

Copy number variation was estimated at the gene level. The pipeline calculated the on-target unique fragment coverage based on consensus bam files, which was first corrected for GC bias, and was then adjusted for probe level bias (estimated from a pooled reference). Each adjusted coverage profile was self-normalized (assuming diploid status of each sample) first and then compared against correspondingly adjusted coverages from a group of normal reference samples to estimate the significance of the copy number variant. Amplification or deletion calls require the absolute z-score and copy number change pass minimum thresholds.

ctDNA fractions were estimated based on the allele fractions of autosomal somatic mutations as described previously (11). Briefly, the mutant allele fraction (MAF) and ctDNA fraction were related as $MAF = (ctDNA * 1) / [(1 - ctDNA) * 2 + ctDNA * 1]$, and so $ctDNA = 2 / ((1 / MAF) + 1)$. Somatic mutations in genes with a detectable copy number change were omitted from ctDNA fraction estimation. ctDNA yields were then estimated by product of total cell-free DNA yield and the fraction of ctDNA.

Plasma-based tumor mutational burden (pTMB) was defined as the number of somatic coding SNVs including synonymous and nonsynonymous variants within panel target regions. The pTMB

score was then normalized by the total effective targeted panel size within the coding region (12).

Analyses of NGS data generated from gDNA

For gDNA sequencing, NGS workflow included preparing the cleaned paired FASTQ files with outputs using in-house workflow pipelines and aligned to human reference genome build hg19 using Burrows-Wheeler Aligner tools. Details of the analytical workflow are provided above in "Analyses of NGS data generated from cfDNA".

Selective/Full Exonic Sequence for the Detection of SNVs, InDels, and CNVs (n=81)									
ABRAXAS1	AKT1	ALK	APC	AR	ARAF	ARID1A	ATM	ATR	BAP1
BARD1	BRAF	BRCA1	BRCA2	BRIP1	CCND1	CCNE1	CD274	CDH1	CDK12
CDK4	CDK6	CDKN2A	CHEK1	CHEK2	CTNNB1	DDR2	DNAJB1	EGFR	EPCAM
ERBB2	ERBB3	ERCC1	ERCC2	ERCC4	ESR1	EZH2	FANCA	FANCC	FANCD2
FANCI	FANCL	FANCM	FBXW7	FGFR1	FGFR2	FGFR3	GNA11	GNAQ	GNAS
HDAC2	HRAS	IDH1	IDH2	JAK2	JAK3	KIT	KRAS	MAP2K1	MAP2K2
MAPK1	MDM2	MET	MLH1	MPL	MRE11	MSH2	MSH6	MTOR	MYC
MYCN	MYD88	NBN	NF1	NFE2L2	NPM1	NRAS	NTRK1	NTRK3	PALB2
PDCD1LG2	PDGFRA	PIK3CA	PMS2	POLD1	POLE	PPM1D	PPP2R1A	PPP2R2A	PRKACA
PRKD1	PTEN	PTPN11	RAD50	RAD51	RAD51B	RAD51C	RAD51D	RAD54L	RAF1
RB1	RECQL	RET	RNF43	ROS1	RPA1	SMAD4	SMO	SPOP	STK11
TERT	TMPRSS2	TP53	TP53BP1	TSC1	TSC2	VHL	XRCC2	XRCC3	XRCC4

Supplementary Table 1. PredicineCare panel targeting 120 genes used in this study.

References

1. Xia S, Kohli M, Du M, Dittmar RL, Lee A, Nandy D, et al. Plasma genetic and genomic abnormalities predict treatment response and clinical outcome in advanced prostate cancer. Oncotarget. 2015;6(18):16411-21.
2. Binder M, Zhang BY, Hillman DW, Kohli R, Kohli T, Lee A, et al. Common Genetic Variation in CYP17A1 and Response to Abiraterone Acetate in Patients with Metastatic Castration-Resistant Prostate Cancer. Int J Mol Sci. 2016;17(7).
3. Zhang BY, Riska SM, Mahoney DW, Costello BA, Kohli R, Quevedo JF, et al. Germline genetic variation in JAK2 as a prognostic marker in castration-resistant prostate cancer. BJU Int. 2017;119(3) 489-495.
4. Huang X, Yuan T, Liang M, Du M, Xia S, Dittmar R, et al. Exosomal miR-1290 and miR-375 as prognostic markers in castration-resistant prostate cancer. European urology. 2015;67(1):33-41.
5. Kohli M, Tan W, Zheng T, Wang A, Montesinos C, Wong C, et al. Clinical and genomic insights into circulating tumor DNA-based alterations across the spectrum of metastatic hormone-sensitive and castrate-resistant prostate cancer. EBioMedicine. 2020;54:102728.
6. Newman AM, Lovejoy AF, Klass DM, Kurtz DM, Chabon JJ, Scherer F, et al. Integrated digital error suppression for improved detection of circulating tumor DNA. Nat Biotechnol. 2016;34(5):547-55.
7. Cibulskis K, Lawrence MS, Carter SL, Sivachenko A, Jaffe D, Sougnez C, et al. Sensitive detection of somatic point mutations in impure and heterogeneous cancer samples. Nat Biotechnol. 2013;31(3):213-9.
8. Xie M, Lu C, Wang J, McLellan MD, Johnson KJ, Wendl MC, et al. Age-related mutations associated with clonal hematopoietic expansion and malignancies. Nat Med. 2014;20(12):1472-8.
9. Genovese G, Kahler AK, Handsaker RE, Lindberg J, Rose SA, Bakhoum SF, et al. Clonal hematopoiesis and blood-cancer risk inferred from blood DNA sequence. N Engl J Med. 2014;371(26):2477-87.
10. Phallen J, Sausen M, Adleff V, Leal A, Hruban C, White J, et al. Direct detection of early-stage cancers using circulating tumor DNA. Sci Transl Med. 2017;9(403).
11. Vandekerkhove G, Todenhofer T, Annala M, Struss WJ, Wong A, Beja K, et al. Circulating Tumor DNA Reveals Clinically Actionable Somatic Genome of Metastatic Bladder Cancer. Clin Cancer Res. 2017;23(21):6487-97.
12. Gandara DR, Paul SM, Kowanetz M, Schleifman E, Zou W, Li Y, et al. Blood-based tumor mutational burden as a predictor of clinical benefit in non-small-cell lung cancer patients treated with atezolizumab. Nat Med. 2018;24(9):1441-8.

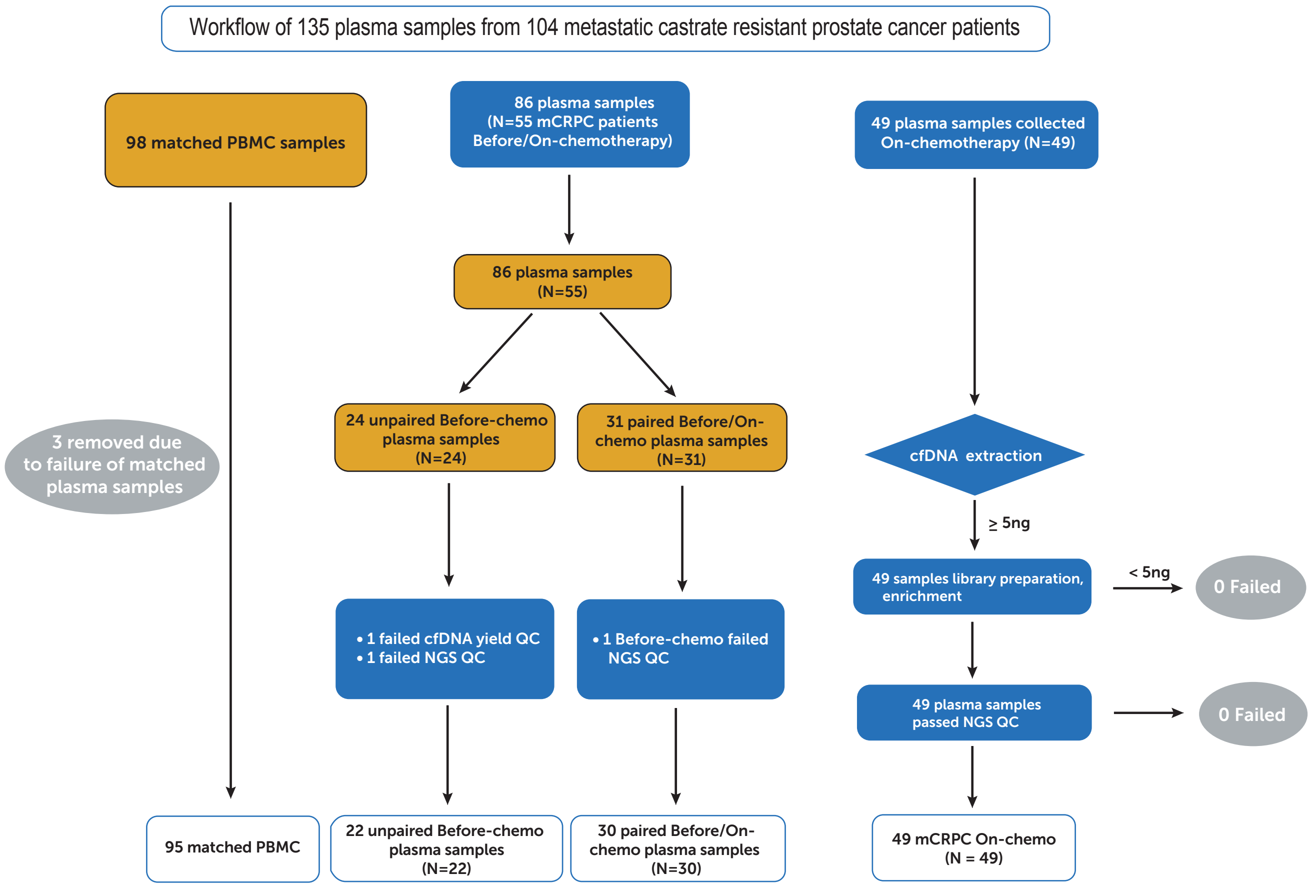

Figure S1. Study design and workflow. This figure was created by manuscript authors using the Adobe Illustrator 2020 software package (https://www.adobe.com/products/illustrator.html).

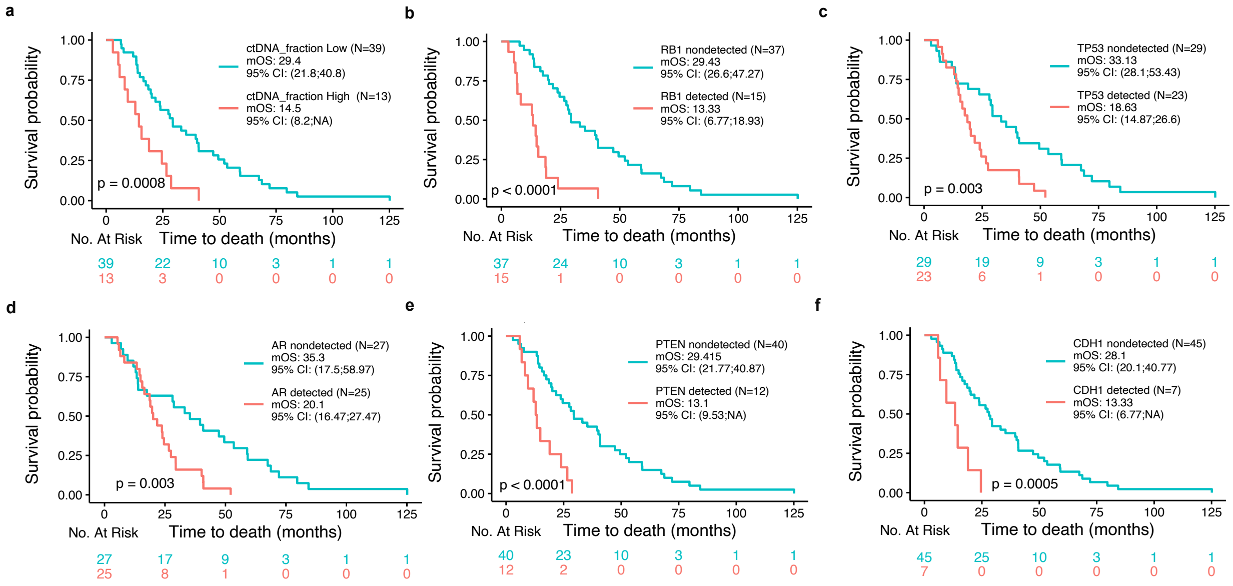

Figure S2. Pre-chemotherapy based somatic alterations and survival. Reduced overall survival (OS) was significantly associated with **a)** high ctDNA fraction (p = 0.0008), and the presence of a SNV or CNV in the *RB1* (p < 0.0001) **(b)**, *TP53* (p = 0.003) **(c)**, *AR* (p = 0.003) **(d)**, *PTEN* (p < 0.0001) **(e),** or *CDH1* (p = 0.0005) **(f)** genes measured in mCRPC patients before the initiation of chemotherapy (n = 52). Survival times were analyzed using the log rank test, with significance set at p ≤ 0.05. The associations between the presence of a SNV or CNV in *RB1*, *TP53*, *PTEN* or *CDH1,* but not *AR*, remained significant after adjustment for age, ctDNA fraction, Gleason Score and alkaline phosphatase levels in multivariate analysis **(Table S2).**

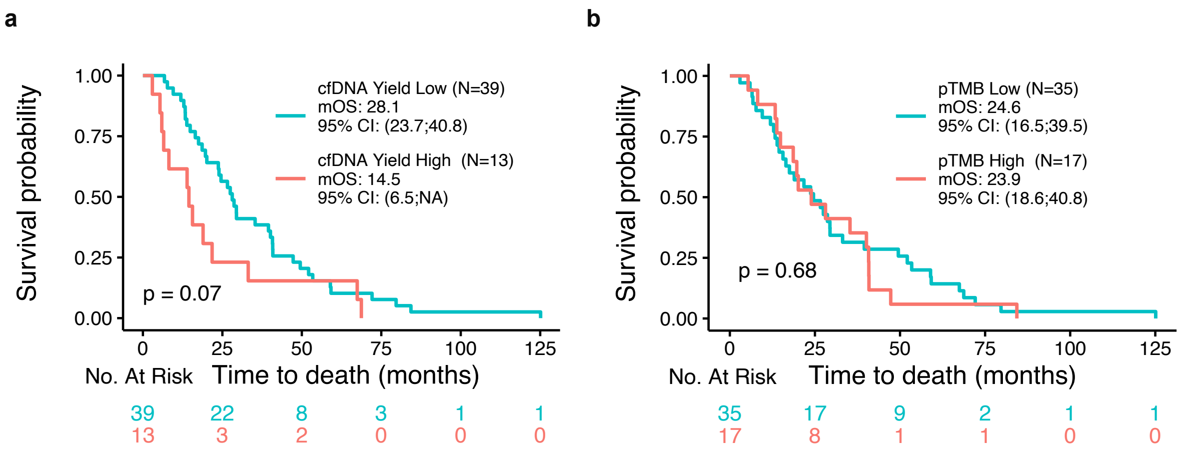

Figure S3. Pre-chemotherapy cfDNA yield, pTMB and survival. Overall survival was not significantly associated with cfDNA yield **(a)** or pTMB **(b)** measured in mCRPC patients before the initiation of chemotherapy (n = 52). The upper quartile was used as a cutoff and difference in survival times was analyzed using the log rank test, with significance set at $p \leq 0.05$.

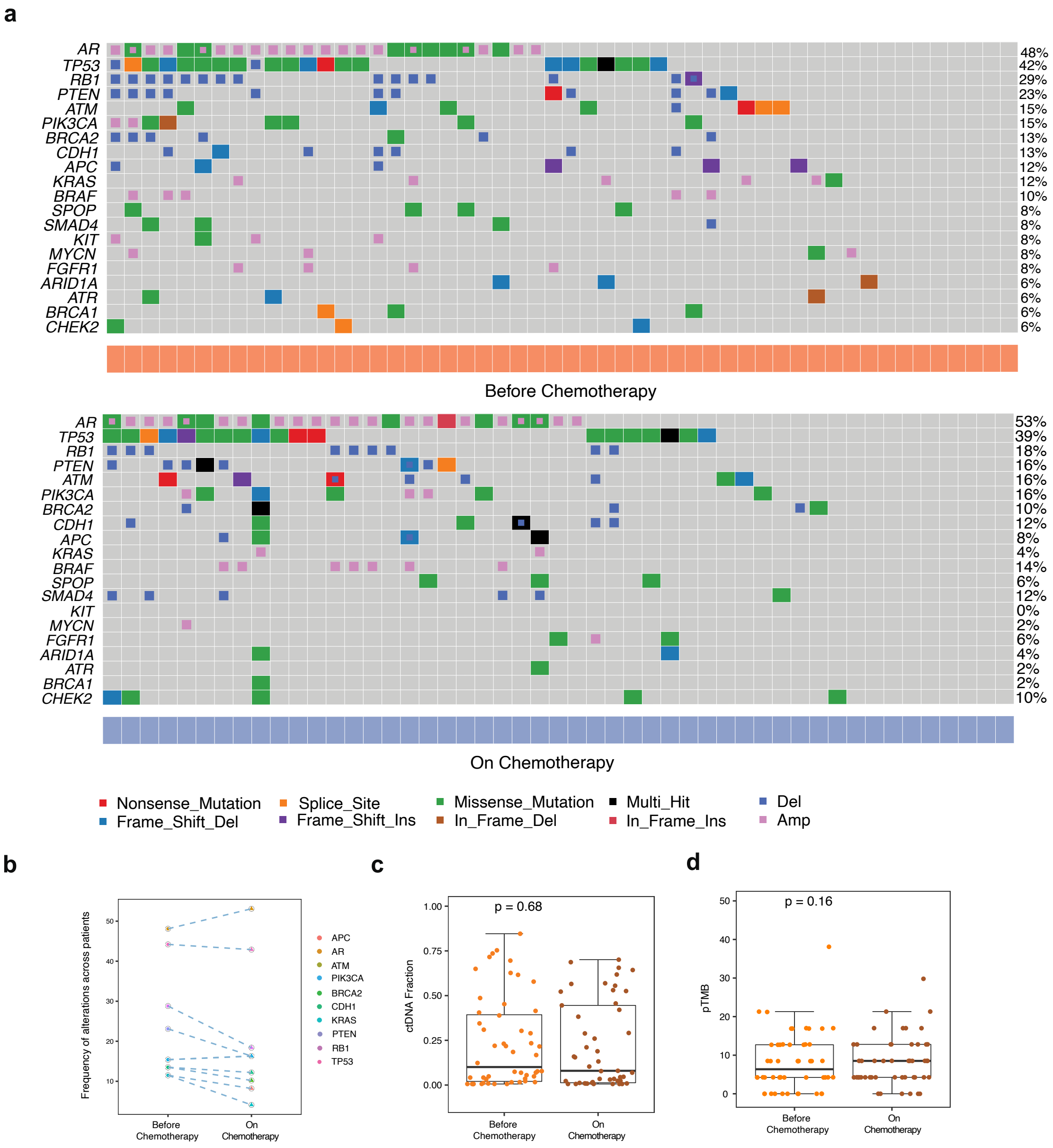

Figure S4. Comparison of ctDNA profiles in the unpaired mCRPC patients sampled before vs. after the initiation of chemotherapy. **a)** Heatmaps of genomic alterations detected before (n = 52) vs. during (n = 49) chemotherapy. No significant differences in profiles were detected between the two independent groups of patients sampled before vs. during chemotherapy with regard to frequency of alterations in the top 10 most frequently altered genes (Fisher's exact test) **(b),** median ctDNA fraction (Wilcoxon test) **(c)**, or median pTMB (Wilcoxon test) **(d)**. The heatmaps in this figure were created by manuscript authors using the Bioconductor ComplexHeatmap software package (version 3.14) (https://www.bioconductor.org/packages/release/bioc/html/ComplexHeatmap.html).

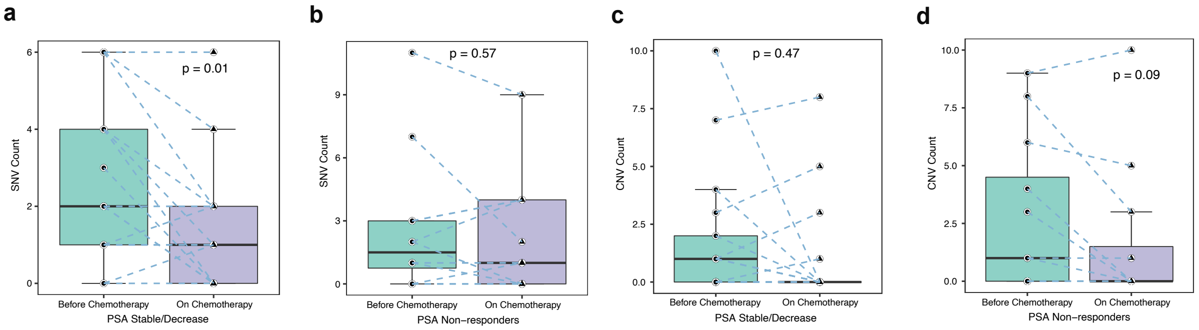

Figure S5. Pharmacodynamic changes in SNVs and CNVs in response to chemotherapy. Comparison of total SNV counts and total CNV counts in paired mCRPC samples collected before and after 3-4 cycles of chemotherapy from patients who were classified for response to chemotherapy on the basis of PSA levels at the time of second sample collection. Significant reductions in total SNV counts were observed after chemotherapy in the patients with stable or decreased PSA levels (PSA Stable/Decrease, n = 17)) (p = 0.01) (**a**), but not in patients with increased levels of PSA (PSA Non-Responders, n = 12)) (p = 0.57) (**b**). In contrast, no significant reductions were observed for total CNV counts following chemotherapy in patients with stable or decreased PSA levels (p = 0.47) (**c**) or in those with increased PSA levels (p = 0.09) (**d**). Comparisons were made with the paired Wilcoxon test with significance set at $p \leq 0.05$.

Gene	Patients with alterations, no. (%)	Median OS, mo	Univariate			Multivariate		
			HR (95% CI)	*P* value	*Adjusted P value*	HR (95% CI)	*P* value	*Adjusted P value*
AR	25 (48.1)	20.1	2.5 (1.3-4.8)	**0.003**	**0.006**	1.7 (0.8-3.7)	0.14	0.22
TP53	23 (44.2)	18.6	2.5 (1.3-4.7)	**0.003**	**0.006**	2.8 (1.3-6)	**0.009**	**0.03**
RB1	15 (28.8)	13.3	4.7 (2.4-9.2)	**1.48E-06**	**1.33E-05**	3.7 (1.4-10.1)	**0.01**	**0.03**
PTEN	12 (23.1)	13.1	4.1 (2-8.7)	**5.75E-05**	**2.60E-04**	3.7 (1.3-10.3)	**0.01**	**0.03**
ATM	8 (15.4)	18.8	1.1 (0.5-2.5)	0.73	0.73	1.1 (0.5-2.7)	0.77	0.86
PIK3CA	8 (15.4)	19.4	1.7 (0.8-3.8)	0.15	0.23	2.6 (1-6.8)	0.05	0.10
BRCA2	7 (13.5)	23.9	1.6 (0.7-3.6)	0.27	0.34	0.9 (0.3-2.6)	0.87	0.87
CDH1	7 (13.5)	13.3	4.2 (1.7-10.2)	**0.0005**	**0.002**	6.3 (1.7-23.6)	**0.007**	**0.03**
APC	6 (11.5)	21.4	1.6 (0.7-3.8)	0.30	0.34	0.7 (0.2-3)	0.68	0.86

Table S2: Univariate and multivariate analyses of overall survival (OS) in association with ctDNA-based alterations. The association of with OS with ctDNA-based alterations (SNV or CNV) detected in the 52-patient cohort (n = 52) prior to the initiation of chemotherapy was evaluated by univariate and multivariate analyses using Cox proportional hazards regression and the log-rank test, with significance set at $p \leq 0.05$. Scaled Schoenfeld residuals and deviance residuals with time were examined to ensure the validity of the Cox regression assumptions. To account for multiple hypothesis testing, adjusted p-values using the Benjamini and Hochberg procedure are reported. Following adjustment for age, ctDNA fraction, Gleason Score and alkaline phosphatase levels, the presence of a SNV *or* CNV in the *TP53* (p = 0.009), *CDH1* (p = 0.007)*, RB1* (p = 0.01) and *PTEN* (p = 0.01) genes remained significantly associated with shorter OS after multivariate analysis.
